# Wearable Triboelectric Sensors Enabled Gait Analysis and Waist Motion Capture for IoT‐Based Smart Healthcare Applications

**DOI:** 10.1002/advs.202103694

**Published:** 2021-11-19

**Authors:** Quan Zhang, Tao Jin, Jianguo Cai, Liang Xu, Tianyiyi He, Tianhong Wang, Yingzhong Tian, Long Li, Yan Peng, Chengkuo Lee

**Affiliations:** ^1^ Shanghai Key Laboratory of Intelligent Manufacturing and Robotics School of Mechatronic Engineering and Automation Shanghai University Shanghai 200444 China; ^2^ School of Artificial Intelligence Shanghai University Shanghai 200444 China; ^3^ Key Laboratory of C and PC Structures of Ministry of Education National Prestress Engineering Research Center Southeast University Nanjing 211189 China; ^4^ Department of Electrical and Computer Engineering National University of Singapore 4 Engineering Drive 3 Singapore 117583 Singapore; ^5^ Center for Intelligent Sensors and MEMS (CISM) National University of Singapore 4 Engineering Drive 3 Singapore 117583 Singapore; ^6^ National University of Singapore Suzhou Research Institute (NUSRI) Suzhou Industrial Park Suzhou 215123 China

**Keywords:** human–machine interface, machine learning, robot‐aided rehabilitation, smart healthcare, triboelectric sensors

## Abstract

Gait and waist motions always contain massive personnel information and it is feasible to extract these data via wearable electronics for identification and healthcare based on the Internet of Things (IoT). There also remains a demand to develop a cost‐effective human‐machine interface to enhance the immersion during the long‐term rehabilitation. Meanwhile, triboelectric nanogenerator (TENG) revealing its merits in both wearable electronics and IoT tends to be a possible solution. Herein, the authors present wearable TENG‐based devices for gait analysis and waist motion capture to enhance the intelligence and performance of the lower‐limb and waist rehabilitation. Four triboelectric sensors are equidistantly sewed onto a fabric belt to recognize the waist motion, enabling the real‐time robotic manipulation and virtual game for immersion‐enhanced waist training. The insole equipped with two TENG sensors is designed for walking status detection and a 98.4% identification accuracy for five different humans aiming at rehabilitation plan selection is achieved by leveraging machine learning technology to further analyze the signals. Through a lower‐limb rehabilitation robot, the authors demonstrate that the sensory system performs well in user recognition, motion monitoring, as well as robot and gaming‐aided training, showing its potential in IoT‐based smart healthcare applications.

## Graphical abstract

A waterproof and textile‐based triboelectric tactile sensor is designed for healthcare applications including motion intention detection for robotic control, human–machine interaction‐based waist training, and gait analysis. Combined with machine learning and Internet of Things technology, all these functions are integrated and demonstrated with a lower‐limber rehabilitation robot to amplify its functionality and improve training outcomes.

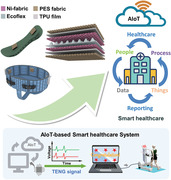

## 1 Introduction

Wearable or flexible electronics emerges as a rapidly developing research field in recent decades because of its intrinsical flexibility and lightness.^[^ ^1^, ^2^ ^]^ It offers a wide range of applications including motion monitoring,^[^ ^3^, ^4^, ^5^ ^]^ rehabilitation,^[^ ^6^, ^7^, ^8^ ^]^ human–machine interface (HMI),^[^ ^9^, ^10^, ^11^, ^12^, ^13^ ^]^ disease diagnosis,^[^ ^14^, ^15^, ^16^ ^]^ etc., to further improve the human's life quality. Among them, various wearable sensors are attached to the skin or worn on the body directly for sensory information collection with promising results distinguishing distinct body behaviors.^[^ ^17^, ^18^, ^19^, ^20^ ^]^ For instance, the smart socks with deep learning‐enabled signal processing and the exoskeleton‐shaped multiple degrees of freedom sensory system were developed for gait analysis and posture perception, respectively.^[^ ^21^, ^22^ ^]^ Such wearable sensory system enables the digitalization of human activities to create data‐based insight into health status and activity management, bridging the gaps between the human and machines. In the meantime, with the remarkable progress of fifth‐generation wireless networks, the delay of the information transmission can be ignored and all the sensory information could be uploaded to the cloud for remote analysis and visualization via Internet of Things (IoT) and big data.^[^ ^23^, ^24^, ^25^ ^]^ Generally, wearable sensors together with seamless data exchange for human motion detection will contribute to smart healthcare applications.^[^ ^26^ ^]^

Disorders of gait and posture are debilitating and common, and adequate monitoring of those disorders is imperative as it can provide useful clues to the underlying pathology in patients and the recovery progress of various medical conditions. Meanwhile, lower‐limb rehabilitation robotic has been proposed as an effective tool to restore the economy of bipedal gait.^[^ ^27^, ^28^ ^]^ For ambulation‐impaired patients, the sensitive and friendly HMI to achieve the dexterous and accurate manipulation of the lower‐limb rehabilitation robot is of vital importance.^[^ ^22^, ^29^ ^]^ Note that the waist always cooperates with the near lower limbs to maintain the walking balance, and provides valuable information of the motion intention. Thus, the HMI devices designed for waist motion capture can provide the sensory information to control the robot to follow and assist the patient's walking. In terms of physical sensing techniques, the vision recognition and initial measurement unit (IMU)‐based HMIs are still the widely adopted solutions to distinguish human motions for robotic manipulation.^[^ ^30^ ^]^ However, the vision recognition by cameras may cause privacy issues, while bulky IMU networks with a lack of flexibility lead to the discomfort.^[^ ^31^, ^32^ ^]^ Furthermore, complicated calculations and high computing power are normally necessary, resulting in sizable external circuits and components.^[^ ^33^, ^34^, ^35^ ^]^ Alternatively, a flexible sensor transferring motion states into electrical signals in a more straightforward way reveals its merits of minimizing the sensory structure. Besides, with the rapid development of virtual reality (VR) and augmented reality (AR), some recent works focus on gaming‐based HMIs via flexible wearable sensors to facilitate the user to absorb in the virtual space.^[^ ^36^, ^37^ ^]^ Such intelligence and functionality‐enhanced HMIs pave a new way to motivate the training with immersive gaming control to relieve the weariness and gain enjoyment during long‐term rehabilitation.^[^ ^38^ ^]^

In terms of flexible wearable sensors, the remarkable process has been made with different structures and materials in the past few years.^[^ ^39^, ^40^, ^41^ ^]^ Till now, several basic approaches are adopted for diversified HMIs and robotic perception, including conductive nanocomposites,^[^ ^42^, ^43^ ^]^ capacitive variation,^[^ ^44^, ^45^ ^]^ piezoelectric or triboelectric effect,^[^ ^46^, ^47^ ^]^ etc. For instance, a cutaneous mechanoreceptor using capacitive variation was developed for soft machines, physiological sensing, and amputee prostheses, showing its outstanding sensitivity and wide pressure range.^[^ ^48^ ^]^ Wang et al. proposed a multifunctional tactile sensor via a hierarchically patterned structure for multiple stimulus detections.^[^ ^49^ ^]^ In general, they exhibit advances in accuracy and stability, but some of the aforementioned methods are still restrained by several drawbacks, e.g., resistive sensor usually suffers the temperature drift and the parasitic capacitance affects the sensing result for the capacitive sensor.^[^ ^50^, ^51^ ^]^ Moreover, the resistive or capacitive sensors must consume considerable electrical power to maintain their operations and they tend to require complicated chemical or physical treatment for fabrication, leading to energy and cost issues for the massive distributed sensory network.^[^ ^52^, ^53^ ^]^ In addition to sensing performance, the flexible wearable sensor is also in high demand of properties including self‐sustainability, ease of fabrication, and low cost for customizable applications.^[^ ^54^, ^55^, ^56^, ^57^, ^58^ ^]^ On that account, the triboelectric nanogenerator (TENG) featured with self‐generated signals and lower power consumption is attracting increasing attention widely, especially for the scenarios of large‐scale IoT applications.^[^ ^59^, ^60^, ^61^, ^62^, ^63^ ^]^ More importantly, due to the unique operation mechanism, triboelectric sensors have versatile options in terms of fabrication techniques and materials.^[^ ^64^, ^65^ ^]^

Textile, as a fundamental part of normal clothes, is well suitable for wearable devices benefiting from its unique properties of light weight, soft nature, and wearable comfortability.^[^ ^66^, ^67^ ^]^ There are a few efforts in developing textile‐based TENG sensors for healthcare applications, which exert superb capabilities of structural retention and fatigue resistance, during long‐term wearing and washing.^[^ ^68^, ^69^ ^]^ For example, He et al. reported a narrow‐gap TENG based on textiles for harvesting low‐frequency and irregular waste energy from body motions to power a Bluetooth module, rendering a self‐sustainable temperature and humidity monitoring system.^[^ ^70^ ^]^ However, such kind of structure integrated with garments is not suitable for robot‐aided lower‐limb rehabilitation in need of gait detection and waist motion capture since the sweat will cause the unexpected performance change during the long‐time on‐body sensing. In consideration of that, the special design for stabilizing the working condition, especially for humidity, becomes extremely important. Note that the thermoplastic polyurethanes (TPU)‐coated fabric usually applied in medical fields like sphygmomanometer features water resistance without the sacrifice of flexibility and thus, this material may provide a promising solution to maintain TENG's performance through facile heat sealing of the TPU layer.^[^ ^71^, ^72^ ^]^ In addition, artificial IoT (AIoT) techniques can be further utilized to amplify the intelligence capabilities of wearable devices via efficient communication with the cloud, and it can extract the higher‐level information from the raw data to simplify the signal processing and compensate the errors.^[^ ^73^, ^74^ ^]^

**Figure 1:**
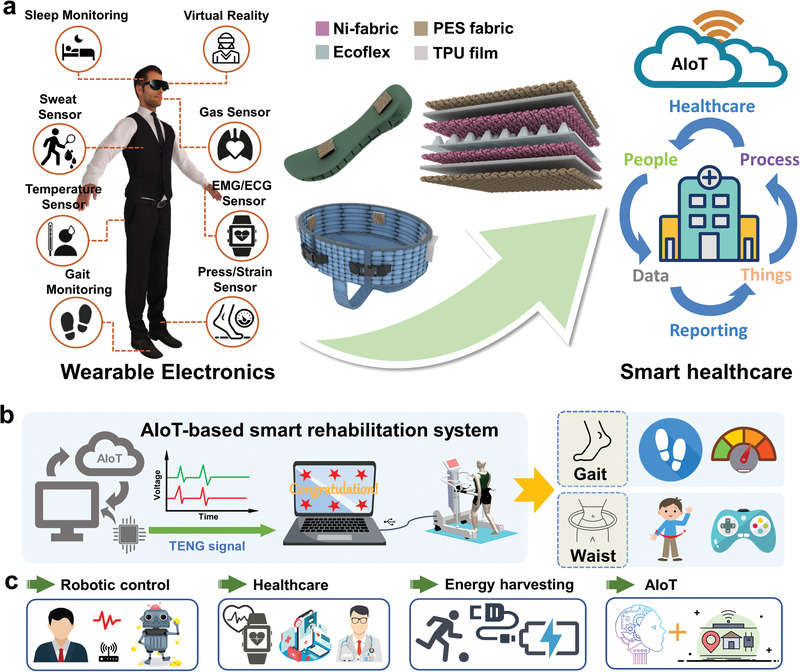
Schematics of the AIoT‐based smart healthcare. a) Wearable electronics including proposed TENG sensors for smart healthcare. b) Schematics of the AIoT‐based smart rehabilitation system with gait detection and waist motion capture. c) Characteristics and future applications of the smart rehabilitation system using TENG sensors.

Herein, we report a textile‐based triboelectric sensory system for gait analysis and waist motion capture to enhance the intelligence and performance of the robot‐aided lower‐limb and waist rehabilitation (**Figure** **1**a,b). Single TENG sensor is structured by the pyramid‐patterned triboelectric layers and it is encapsulated by TPU‐coated fabric via heat sealing to ensure performance stability. Four such triboelectric sensors are equidistantly sewed onto a safety belt designed for supporting the user, enabling waist motion recognition. The relative TENG sensors will be squeezed and they generate distinct triboelectric signals depending on waist motions. Based on the waist TENGs (W‐TENGs), the real‐time robotic manipulation and virtual game are introduced for immersion‐enhanced training with the real‐time HMI. The insole equipped with two TENG sensors is designed for gait detection, i.e., walking steps, speed, and status to record the training process. Leveraging the diversified information from insole TENGs (I‐TENGs), the machine learning technique is utilized to analyze the signals to realize user recognition and rehabilitation plan selection for protecting privacy. We further integrate the smart insole and safety belt into a lower‐limb rehabilitation robot (iReGo) to demonstrate the sensory system capable of user recognition, motion monitoring, robotic manipulation as well as gaming‐enhanced training. In general, our triboelectric rehabilitation device provides a low‐cost, energy‐saving, and universal solution for the realization of the AIoT‐based smart healthcare system in near future (Figure 1c).

## 2 Structure, Working Principle, and Characterization

**Figure 2:**
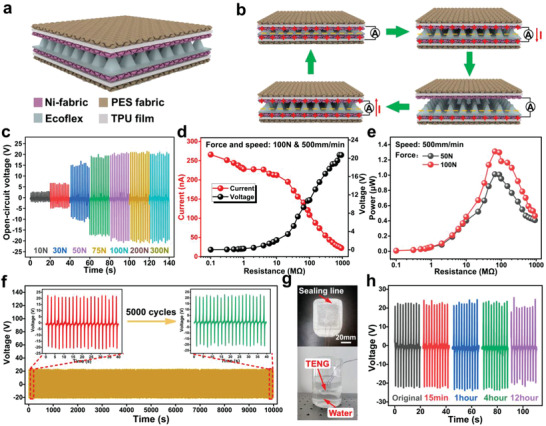
Characterizations of the textile‐based TENG sensor. a) Structure and b) working mechanism of the TENG sensor. c) Open‐circuit voltages of the TENG sensor with different compressing forces. d) Output characterizations of the TENG sensor with 100 N compressing force and 500 mm min^−1^ compressing speed. e) Power curves of the TENG with 50 and 100 N compressing forces. f) Output voltages during the cyclic compressing test. g) Images of TENG sensor and its dipped‐in‐water status. h) Output characterization of the TENG after dipping in the water.

Though the TENG technology offers a facile and low‐cost fabrication process for flexible wearable sensors, the sensitivity and sensing range are required to be considered since there always suffer distinct pressures during monitoring gait and waist motion. Also, building up a water‐resistant structure is of vital importance for the scenarios of long‐time and high‐intensity training as the sweat will lead to voltage variation. Here, we adopt the encapsulated narrow‐gap structure with pyramid patterns distributed on the negative triboelectric layer to construct the device (**Figure** **2**a). The TENG sensor is basically composed of five layers, where two TPU‐coated polyester (PES) fabric layers wrap the inner triboelectric layers and electrodes to maintain the humidity without the sacrifice of flexibility. The negative triboelectric material is a thin silicone rubber layer and a conductive nickel fabric (Ni‐fabric) coated on its backside that serves as the electrode. To create the pyramid pattern, the silicone rubber is fabricated by a 3D‐printed mold with special concaves (Figure S1, Supporting Information). Another Ni‐fabric is arranged to contact the negative material face to face so that the charge transfer occurs due to the contact‐separation mode based on the working mechanism of the TENG sensor (Figure 2b). Thus, the pressure stimulus will generate the voltage output directly and it is able to harvest the mechanical energy into electricity via certain external circuits to achieve self‐powered sensing and rehabilitation applications.

To characterize the electrical output performances of the TENG‐based sensor, a multi‐material test machine is employed to adjust the compressing pressure and speed (Figure S2, Supporting Information). The 35 mm × 35 mm triboelectric sensors with the same pyramid number but different sizes are used to validate the sensitivity (Figure S3a, Supporting Information). As depicted in Figure S3b in the Supporting Information, with the same height (3 mm), the smaller pyramid size (3 mm × 3 mm) possesses the largest output under 100 N compressing force compared to other devices with a larger pyramid or none‐pyramid structure. Additionally, although it seems that output can be further enhanced by decreasing the pyramid size, but there remains a challenge to mold the structure because of the air bubble. We further characterize the TENG sensor with different positive triboelectric materials and the result validates the conductive textile (Ni‐fabric)‐based TENG shows the best performance (Figures S3c and S4, Supporting Information). Eventually, the as‐fabricated sensor with optimal sensitivity (i.e., 3 mm × 3 mm pyramid pattern) is selected to verify the distinct application scenarios (Figure S5, Supporting Information). We exert different compressing forces ranging from 1 to 300 N to squeeze the triboelectric sensor and the output properties are investigated to understand the response to different normal forces. As shown in Figure 2c and Figure S6 in the Supporting Information, the output rises along with the increase of forces. The sensor seems to approach the saturated state after the compression force is larger than 100 N and the peak voltage variation under 100 to 200 N is quite small. Meanwhile, the 1 N compressing force generates 0.7 V output and when it comes to 100 N, the voltage reaches 20 V, showing that the voltage‐sensitive range is from 0.8 kPa to more than 81.6 kPa. Normally, for gait and waist motion monitoring, there requires the sensor to work under about 10 and 120 kPa, respectively. Our experiment validates that it is feasible to generate signals for TENG under more than 300 N, i.e., 245 kPa, reflecting that the TENG sensor is suitable for both applications (Figure 2c).

Considering the TENG‐based sensor also has grand potential in energy harvesting, its output performance is measured with various external load resistances from 0.1 to 1000 MΩ (Figure 2d). Accordingly, it can be found that the output voltage of the device increases monotonously to 20 V with a 1 GΩ resistance and the corresponding current decreases based on Ohm's law. When the device is tested under 0.5 Hz and 100 N, the maximum power is about 1.3 µW with an external resistance of 90 MΩ (Figure 2e). Meanwhile, the curve of 50 N loading force exhibits a similar trend, whereas the maximum power changes to about 1 µW. Subsequently, we investigate the influence of the pushing speeds on the output performance. As shown in Figure S7 in the Supporting Information, regardless of the applied force (50 or 100 N), although the transferred charges seem to be identical, both the open‐circuit voltage and short‐circuit current increase with a higher compressing speed. In general, the proposed textile‐based TENG sensor is capable of energy harvesting from the cyclic stimuli. To demonstrate its self‐powered property, 55 commercial light‐emitting diodes are lighted without external power after tapping the device to charge the capacitor (Figure S8, Supporting Information). As for rehabilitation, it always represents the repeated and long‐term training so that the sensing component should be eligible for the long service time. Hence, the fatigue test is implemented to claim that the sensor maintains the same output even after more than 5000 cycles, as shown in Figure 2f. In addition, the sweat or dust during intensive training always affects the working conditions, e.g., material performance and humidity, leading to unstable performance. Owing to the TPU film‐based sealing design, as depicted in Figure 2g,h, our device exhibits its super water‐resistant characteristic after dipping in the water for more than 10 hours, proving the washability and stability to act as an on‐body wearable device. In the meantime, we also demonstrate that the output of TENG remains a constant value with 20 to 80 ℃, further showing its high robustness (Figure S9, Supporting Information). In summary, the proposed sensor with good sensitivity, signal robustness, water resistance, and temperature stability makes a solid promise to achieve self‐powered sensing in smart healthcare applications.

## 3 TENG‐Based HMI for Waist Training

**Figure 3:**
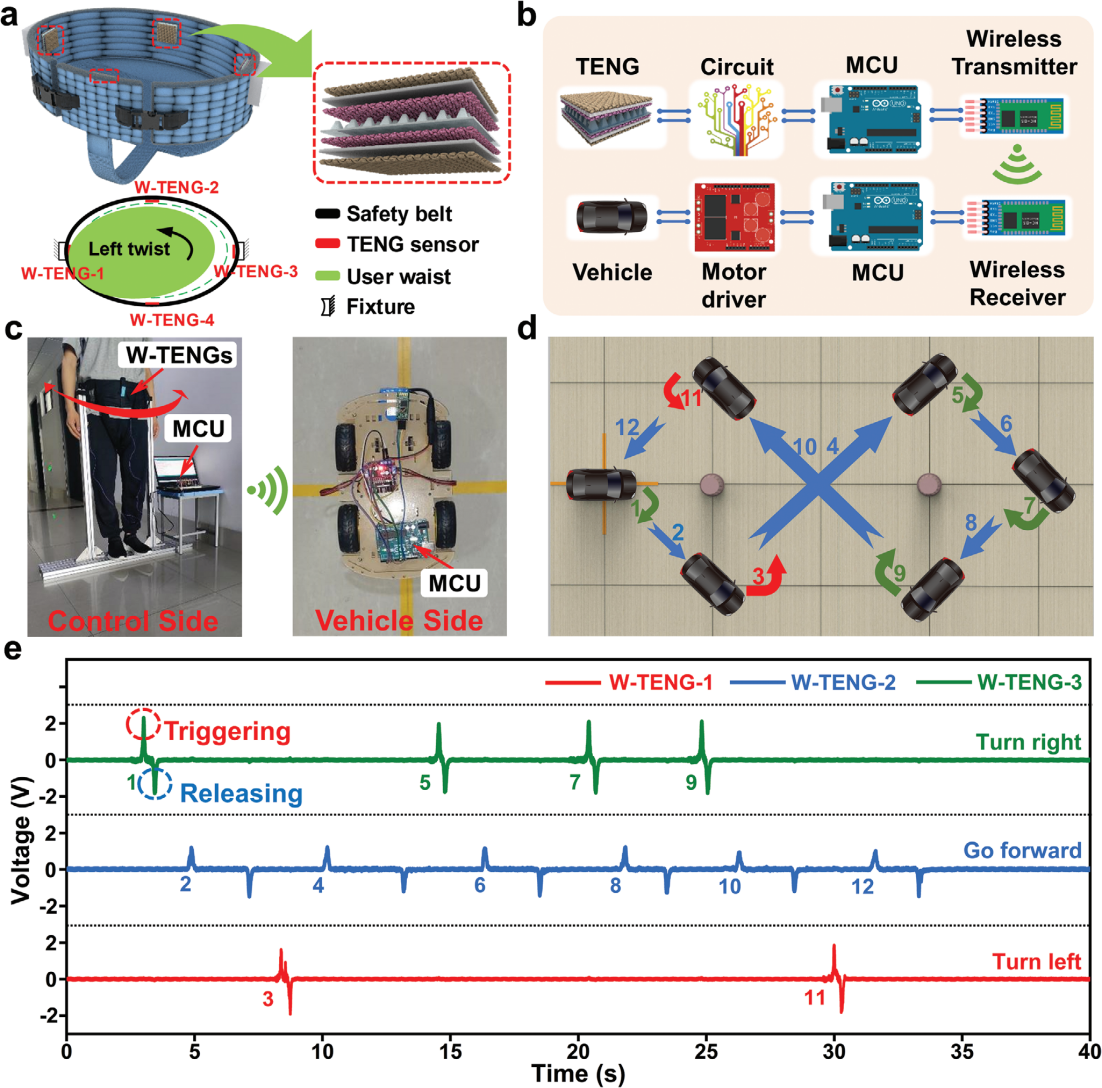
HMI enhanced waist rehabilitation via intelligent safety belt. a) Schematics and working mechanism of the intelligent safety belt with four TENG sensors marked as W‐TENG‐1 to W‐TENG‐4. The safety belt is fixed to the fixture similar tothe rigid bracket of the rehabilitation robot. b) Hardware and flowchart for real‐time vehicle control. c) Images of experimental setup using the safety belt with TENGs to control the vehicle. d) Schematics and h) real‐time signals to control the vehicle by W‐TENGs, where motions and related signals are marked by the same number.

In modern society, with the rapid development of technology, intelligent machines tend to replace heavy physical labor and people start to break away from the traditional work style. Alternatively, they remain seated and immobile staring at computer monitors for a long time, leading to the high risks of waist injuries.^[^ ^75^ ^]^ The device for waist rehabilitation becomes significantly important because the health of the waist is one of the major factors to maintain the life quality. However, weariness and inattention hinder the long‐term waist training, and there remains a demand to improve the enjoyment and motivation for a better rehabilitation outcome. Meanwhile, benefiting from the drastic advancement of AR/VR technologies, the promotion of diversified HMIs paves a new way for immersive healthcare programs via the virtual game, digital twin, robotic manipulation, etc.^[^ ^76^, ^77^ ^]^ Hence, in order to construct the HMI for waist rehabilitation apparatus, four textile‐based TENG sensors are sewed onto the waist safety belt equidistantly to form W‐TENGs. As depicted in **Figure** **3**a, the safety belt is usually fixed to the robot or fixture to support the user. Owing to the distributed arrangement, the W‐TENGs marked as W‐TENG‐1 to W‐TENG‐4 will be activated in response to the related waist motions, e.g., the left twist will generate the voltage signal of W‐TENG‐1. Based on this working mechanism, it is possible to realize the motion intention detections such as going forward/back, turning right/left by distinguishing the signals. Here, we further demonstrate the W‐TENG to serve as the HMI for robotic manipulation through a real‐time vehicle control system to validate the sensing mechanism. Hardware and flowchart for the remote vehicle manipulation can be found in Figure 3b, where the microcontroller unit (MCU) with the related circuit (Figure S10, Supporting Information) and Bluetooth module is used to process the signals. After the signals generated from different W‐TENGs are detected by MCU, the corresponding command will be transmitted via the wireless Bluetooth module simultaneously to realize the vehicle control.

Based on such sensory method, the intelligent safety belt‐based HMI together with rigid bracket to support the user is implemented to realize waist rehabilitation enhanced by vehicle control, as shown in Figure 3c. In this demonstration, three TENG sensors marked as W‐TENG‐1 to W‐TENG‐3 are applied. As depicted in Figure S11 in the Supporting Information, when the volunteer on the control side tends to stretch forward, twist left or right the waist, the positive triboelectric voltage occurs to lead the vehicle to exert the related response, validating the feasibility of TENG‐based control. Moreover, the negative signal peak will be generated along with the volunteer recovering to the original state and it can be harnessed to change the controlled target into the static state. Afterward, this sensory system is implemented to follow a set of movement routes to verify the HMI for waist rehabilitation training, where the differently colored arrow represents motion directions, i.e., red, green, and blue arrows for turning left, turning right, and moving forward, respectively (Figure 3d). Besides, the order of execution is numbered along with the arrows to match with the real‐time triboelectric signal in Figure 3e, where the triggering and releasing signals (i.e., positive and negative signals) start and stop the vehicle's movement in the time sequence. As a result, the safety belt‐based HMI controls the manipulation and the vehicle passes the obstacles with the 8‐shaped trajectory (Video S1, Supporting Information). In general, this approach plays a key role in either training the waist or monitoring the mobility of disabled patients in the rehabilitation process. Moreover, it reveals the potential for a more complex HMI enhanced by AR/VR technologies for robot‐aided rehabilitation applications.

## 4 Smart Insole‐Based Gait Analysis

**Figure 4:**
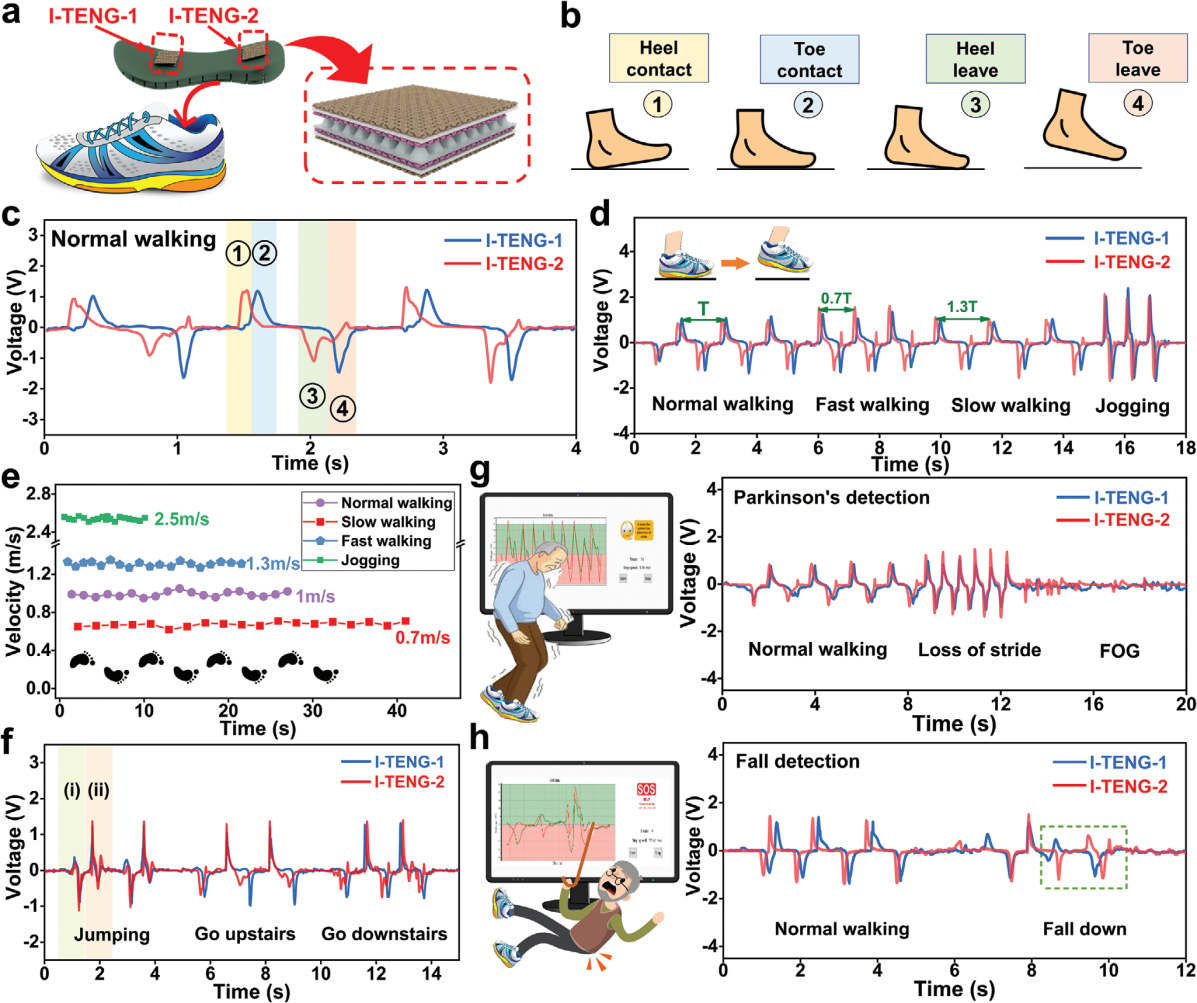
TENG‐based smart insole for gait analysis. a) Structure of the insole with two TENG sensors marked as I‐TENG‐1 and I‐TENG‐2. b) Schematics of four states of a typical contact cycle and c) the corresponding signals of normal walking. The walking states and associated signals are marked with the same number. d) Voltages of different walking modes, where the foot is on the ground at the original state. e) Real‐time walking speed detection based on the signal frequency. f) Voltages of jumping, going upstairs, and going downstairs, where the state (i) and state (ii) for energy storage and landing during jumping, respectively. g) TENG‐based smart insole to detect Parkinson's symptoms and h) to detect fall. FOG represents freezing of gait.

Gait always contains valuable sensory information and it makes sense to monitor the gait via distinct sensors to collect the diverse physical parameters.^[^ ^78^, ^79^ ^]^ For instance, in items of the robot‐aided lower‐limb rehabilitation, as the patient takes walking training, the necessary information can be digitalized to explain the step number, walking speed, etc., enabling to regulate the amount of training and analyze the rehabilitation status. Currently, two basic concepts are adopted to accomplish this task, i.e., sensitized insole or sock.^[^ ^80^, ^81^, ^82^ ^]^ In this work, a couple of textile‐based TENGs marked I‐TENG‐1 and I‐TENG‐2 are fabricated and arranged onto the front and back sides of the insole, respectively, to improve the intelligence for gait analysis, as shown in **Figure** **4**a. During a single walking cycle, the process can be divided into four states, i.e., “heel contact,” “toe contact,” “heel leave,” and “toe leave” in Figure 4b. Based on the working principle of TENG, both the I‐TENGs on the insole will be continuously squeezed and released to generate the triboelectric signals. To validate this phenomenon, one volunteer (175 cm and 75 kg) conducts normal walking by wearing the shoes integrated with the smart insole. As depicted in Figure 4c, when the user walks forward, two positive peaks for the heel contact and toe contact are observed during the half‐cycle of gait, and two negative peaks associated with heel leave and toe leave are detected thereafter. Noticeably, all the signals in Figure 4c can match with the walking states in Figure 4b and the same number are used to emphasize this feature. Besides, compared to the contact force in Figure S12 in the Supporting Information, the signal curve in Figure 4c seems to be smoother because of the signal processing circuit and sensor's performance. Eventually, the user's gait can be further detected and analyzed through the characteristics extracted from the voltage value and frequency. More specifically, the peak number of the voltage signals can be harnessed to count the step number by setting a threshold value and the time intervals between the peaks of the voltage signals representing signal frequency are useful for sensing the walking speed.

To further verify the reliability of smart insole‐based gait analysis, some extra experiments have been conducted by the same volunteer. As shown in Figure 4d, the TENG sensors are successfully activated whether in the normal walking mode, fast walking mode, slow walking mode, or jogging mode. It can be found that the voltage for jogging is apparently larger than the walking and thus it can be further utilized for walking mode detection. Moreover, because of the compressing time and impact force, there also occurs the voltage variation for walking at different speeds and it seems that the fast walking exhibits the largest output while the comparably smaller one for slow walking. Besides, the time intervals between the two positive voltage peaks are related to the speed and the time interval for fast walking (≈0.91 s) is the smallest compared to normal walking (≈1.3 s) and slow walking (≈1.69 s). We also verify the real‐time walking speed and it is tested by matching spots on the ground to walk 30 m (Figure S13, Supporting Information). Thus, the walking speed can be obtained by the equation *V*_s_ = *L*/*T*, where *T* is the time interval between two positive voltages for heel contact, and *L* is the stride of one walking cycle. As shown in Figure 4e, although there exist some speed fluctuations to complete the same distance, the detected speed matches with the average speed well for all the walking modes. Eventually, the experiment to demonstrate real‐time walking speed and status detections with an interface is further implemented to validate the functionality and feasibility of the smart insole (Video S2, Supporting Information).

To explore the signals except for four‐state walking on the flat ground, the TENG signals for jumping, going upstairs, and going downstairs are measured. As shown in Figure 4f, unlike normal walking with sole contact and separation, the jumping suffers more complicated signals, which can be divided into state (i) and state (ii). The reason for this phenomenon is that the jumping undergoes energy storage and landing steps, and the foot leaves and contacts the ground at the same time. Meanwhile, signals for going upstairs or downstairs possess their specific characteristics as well, e.g., the heel and toe contact at the same time for going upstairs and the toe tends to stand first to ensure safety for going downstairs. Depending on the sensitivity of the smart insole, we apply it for Parkinson's disease (PD) and fall detection to alarm for the emergency situation during the rehabilitation process. PD is a progressive neurodegenerative movement disorder that often manifests as gait disturbances and even falling. However, PD is difficult to be evaluated during clinical examination because it is usually triggered by uncertain or specific conditions, resulting in the significance of monitoring patients with PD. In addition, for these elderly patients or the disabled, the capability of fall detection is of vital importance to guarantee human life. To reflect the possibility to utilize the smart insole in these applications, the mimetic motions of falling and PD are performed, and the corresponding detected signals can be found in Figure 4g,h. For PD, three states of normal walking, loss of stride, and freezing of gait (FOG) are sequentially monitored during the mimetic experiment. As shown in Figure 4g, patients usually accelerate their step frequency involuntarily after the loss of stride, while they become unable to move their feet even if they intend to do so during FOG, leading to the unique output signal, i.e., the signal frequency rises suddenly before the messy signal occurs. Besides, when the patient is walking normally and suddenly falls, the voltage signal appears two compact negative peaks and no more positive peaks thereafter, indicating that the user's foot remains off the ground (Figure 4h). Herein, as shown in Video S3 in the Supporting Information, both emergency events are recognized by the gait monitoring system and it generates the alarm immediately. Generally, these demonstrations show the great potential of our TENG‐based insole for smart healthcare applications, especially in real‐time remote health monitoring and emergency alarm for the elderly and disable patients during rehabilitation training.

## 5 Machine Learning for Patient Recognition

In this era of information expansion, there arises the privacy concern of the face, fingerprint, and acoustics recognitions because of their massive personnel information.^[^ ^83^ ^]^ Gait implicates multi‐layer characteristics (e.g., weight and walking habit) and these privacies always couple together to form facile and natural encryption via a set of triboelectric signals using the proposed smart insole. Unlike traditional analysis strategies to obtain the shadow features such as frequency and peak number, recent emerging machine learning as a technique for extracting nuances has been used for data acquisition and pattern analysis of triboelectric signals, providing the possibility of integrating artificial intelligence (AI) technology with wearable electronic technology to construct a complete intelligent system.^[^ ^84^ ^]^ Moreover, this approach can comprehend subtle information for complex features in a higher dimension which cannot be resolved by naked eyes after training the model. Among the machine learning methods, artificial neural network (ANN) is a quite effective approach to solve complex classification problems with good robustness and scalability, and it has been proposed to be applied in analyzing tactile signals for high‐accuracy object recognition.^[^ ^85^, ^86^ ^]^ Thus, the smart insole combined with ANN‐based predicting model provides an alternative solution for patient recognition to fulfill the scenarios involving information identification, avoiding the risks of privacy disclosure.

**Figure 5:**
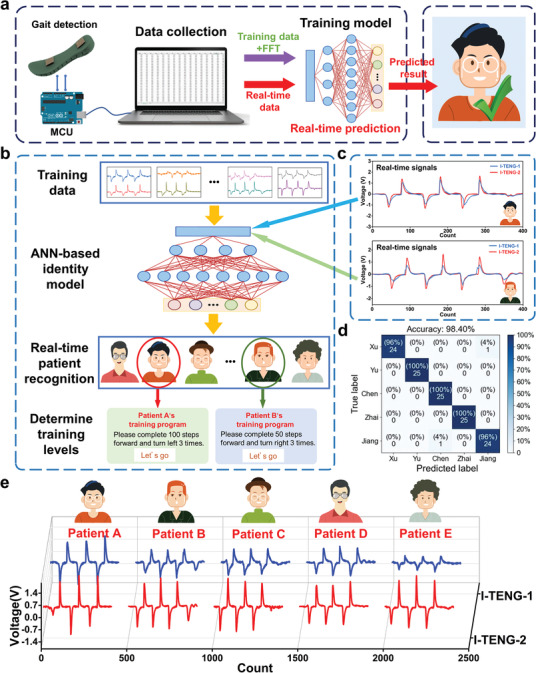
Patient identification based on the smart insole and machine learning. a) Schematic diagrams of the patient recognition by smart insole and machine learning. b) Overview of the recognition system. c) Real‐time signals for different patients. d) Confusion map of the machine learning training result. e) 3D plots of the I‐TENG sensor outputs corresponding to five patients, where the five participants in (d) are marked as patient A to patient E.

Herein, we apply ANNs integrated with the fast Fourier transform (FFT) to extract features from the triboelectric signal to realize the patient recognition for rehabilitation plan selection (**Figure** **5**a,b). To build up the machine learning model, five participants with different weights ranging from 55 to 75 kg are volunteered to perform the same stepping action as they wear shoes with the smart insole. During the test, analog voltage signals generated in the TENG‐based smart insole are first collected and processed by the hardware circuit consisting of an ADC and MCU. Subsequently, the signals of both the I‐TENG‐1 and I‐TENG‐2 sensors are recorded for 150 times of tests (three steps each time) to acquire the dataset, while a specific window to visualize the 400‐point length signals are utilized to ensure the effectiveness of the collected data, i.e., each sample should contain the sensory information for three continuous steps, as shown in Figure 5c. Noticeably, although a larger dataset always represents more features for a higher recognition accuracy, we only collect the sensory information for the right foot to decrease the amount of data to simplify the system, exhibiting the trade‐off between sample number and accuracy. Then, we use the row voltage data of two channels directly as the features of samples, so there are 400 × 2 = 800 features for each sample and each feature represents one data point at time series during stepping, meaning that the data include the information of the contact force, stepping speed, contact duration, etc. Figure 5e presents the typical outputs of sensors generated from five different participants and all the 150 samples of each participant are processed via FFT to eliminate the time‐domain information but remain the frequency characteristics before the samples are randomly split into three groups at the ratio of 4:1:1 (training: 100 samples, testing: 25 samples, and validation: 25 samples). Afterward, all the selected 100 samples are directly input to the ANNs framework to establish the predicting model. Noticeably, the proposed network architecture with different fully connected layers (FCLs) is utilized to optimize the trained model under the evaluation of the cross‐entropy loss function.^[^ ^87^, ^88^ ^]^ Meanwhile, we insert dropout and batch normalization layers into the first several FCLs to enhance the robustness and accelerate the training speed, while the last FCL outputs the predicted identifiers of the five participants (the five‐FCL architecture can be found in Figure S14, Supporting Information). Our result reflects that the architecture consisted of five FCLs exhibits the best performance (i.e., the smallest value for loss function) with the identical epoch of training and its accuracy of training set reaches 100% after ten epochs (Figure S15, Supporting Information). Eventually, the five‐FCL ANN‐based model is validated to possess the high positive predictive value and true positive rate for patient recognition, and as shown in Figure 5d, the total recognition accuracy reaches 98.4%.

In terms of the healthcare system, the expensive medical devices such as the rehabilitation robot are frequently shared by multiple patients and there usually occurs privacy issues as the training data including personal information are always left in the machine, bringing in the motivation for patient recognition and constructing accessible private accounts in a feasible and user‐friendly manner. Based on the machine learning model, we extend the identity recognition into the rehabilitation system, and thus, the customized training plan can match with different patients via the IoT‐based distant setting. Note that the sole 100‐sample training data are proved to realize a high recognition accuracy, indicating the significance and possibility for the disabled patient to follow this identification. We demonstrate the patient recognition based on the established model for the same five participants and the result indicates that the system accomplishes the function to access their personal accounts for training plan selection (see Video S4, Supporting Information). The establishment of such an intelligent system can improve the efficiency of rehabilitation training and facilitate communication among the doctor, machine, and patient, providing the possibility of remote diagnosis and home rehabilitation.

## 6 Robot‐Aided Rehabilitation Applications

As the elderly population increases, the rehabilitation robot to assist disabled patients to recover from the diseases like stroke and muscle atrophy is in great demand to improve the life quality. iReGo, a kind of lower‐limb rehabilitation robot, originally possesses a rigid bracket with a safety belt to support the patient and a motor‐driven structure to assist walking, making a promise for walking and waist training (Figure S16, Supporting Information).^[^ ^89^, ^90^ ^]^ Although it already has a robotic control system based on the potentiometer and electric switch, these components require a bulky rigid mechanism to realize the function, leading to extra resistance and uncomfortability during rehabilitation. Furthermore, they always lack sensitivity because of the inevitable structural clearance. Frankly, for the robot‐aided healthcare program, comfortability and interest are crucial to improve the immersive experience for better training outcomes since motivation affects the long‐term training a lot. In the meantime, the aforementioned self‐powered TENG‐based wearable sensor is proved to act as the sensitive monitoring sensor, showing its potential in HMIs for robotic manipulation as well as gaming‐enhanced training. Herein, we integrate both the intelligent safety belt and smart insole into commercially available iReGo to validate the advancement of the triboelectric sensory system.

**Figure 6:**
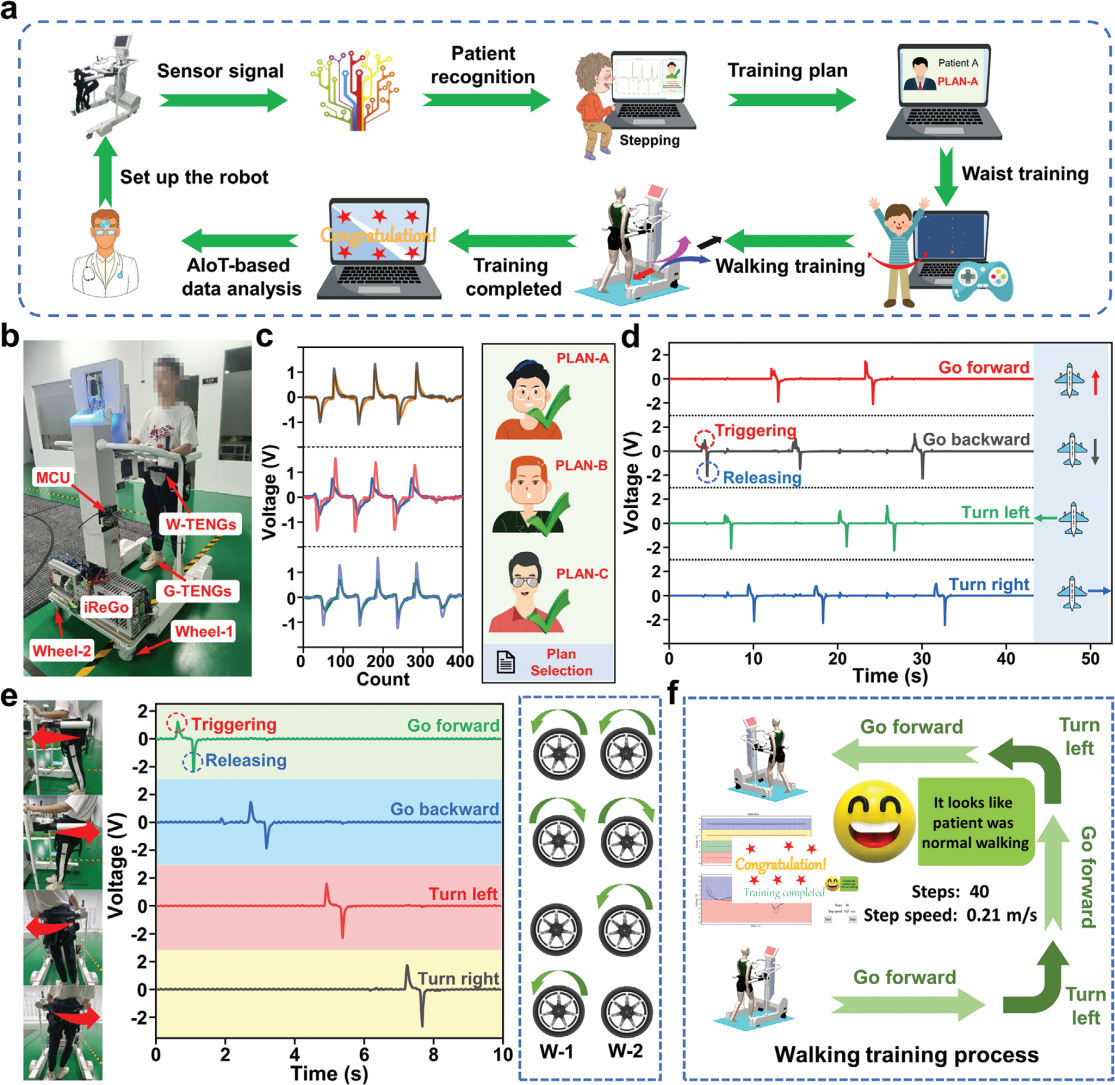
Demonstration of the robot‐aided rehabilitation with triboelectric sensory system. a) Schematics of the robot‐aided rehabilitation process. b) Configuration of the lower‐limb rehabilitation robot (iReGo) integrated with the TENG sensory system. This robot possesses two motor‐driven wheels and a safety belt to support and guide the walking training. c) Diagrams of training plan selection based on patient recognition. d) Gaming‐enhanced waist training using TENG‐based safety belt for improving the enjoyment. e) Demonstration of rehabilitation robot control with TENG‐based safety belt to help patient walk. f) Diagrams to illustrate the walking training process.

The process flow of robot‐aided rehabilitation based on triboelectric sensory system is depicted in **Figure** **6**a, dedicating to link patients, machines, and doctors to improve intelligence. Figure 6b illustrates the iReGo integrated with the intelligent safety belt and smart insole, where the TENG sensors are sewed onto the robot's safety belt and the smart insole is utilized directly. After performing the stepping to the smart insole, the robot is able to make an identity judgment to ensure privacy and offer the individual training plan. Among the identification, the deep learning techniques are applied and three different users are verified for identification with the smart insole. As shown in Figure 6c, distinct users bring different TENG signal maps and thus different training schedules can match with the user directly. Hence, the system can pop up the personal training amount set by the doctor in advance according to the user's physical condition before the lower‐limb training (see Video S4, Supporting Information). Then, combined with a four‐TENG‐based safety belt, a virtual game named plane wars is introduced to utilize TENG signals to enhance the immersion for increasing the interest and motivation during waist training. Also, it is possible to develop the AR/VR‐enhanced game following a similar method, providing the possibility of wider HMI scenarios. As depicted in Figure 6d, the positive peak corresponding to compressing the TENG sensor triggers different movement directions of the plane, while the negative signal after releasing the TENG sensor is used for stopping the movement. Here, the movement direction is also related to the TENG arrangement the same as Figure 3a. A video showing real‐time gaming‐enhanced waist training can be found in Video S5 in the Supporting Information, where the waist motion is recorded until reaching the training target. Similarly, after finishing the waist training, we further apply the safety belt‐based HMI to realize the robot‐aided walking training. As depicted in Figure 6e, due to the robot's rigid bracket, different waist movement intentions render different TENG sensors to be squeezed and they generate different signals to control the motion of different wheels. For instance, the signals trigger two wheels to rotate forward as the user squeezes the front TENG and vice versa. Furthermore, when the left or right TENG is activated, the robot's two wheels operate in the reverse direction to turn left or right. Here, a video (Video S6, Supporting Information) is used to demonstrate the introduced robotic manipulation applying both iReGo and intelligent safety belt. In addition to patient recognition, the smart insole is further harnessed to record the gait during walking training, ensuring the training safety and rehabilitation status prediction. Therefore, according to the training plan, the interface to display the walking training situation is developed to monitor walking speed, step, and status (Figure 6f and Video S7, Supporting Information). After completing the training amount, the system reminds the user that the training is over and relevant information will be sent to the doctor for training evaluation. In short, such a reasonable and effective training system is able to achieve immersive lower‐limb rehabilitation with multiple functions including patient recognition, robot‐aided and gaming‐enhanced training, and distant diagnosis, making a good promise for IoT‐based smart healthcare.

## 7 Conclusions

In summary, a wearable triboelectric sensory system has been developed for digitalizing human motions to enhance the intelligence and performance of lower‐limb and waist rehabilitation. The wearable sensory system consists of a smart insole for gait analysis and an intelligent sensory safety belt to detect waist motions, both equipped with textile‐based sensing elements on the basis of triboelectric effect. The textile‐based TENG sensor composed of pyramid‐patterned triboelectric layers is encapsulated by sealing TPU‐coated fabric to expand the sensing range as well as maintain its output property regardless of the humidity condition and even sweat, making it more suitable for long‐term gait analysis and waist motion capture. To track the movement of waist comprehensively for robot‐aided rehabilitation, the safety belt integrated with four triboelectric sensors is designed to output distinct signals according to waist motions. The sensory belt is used to realize AR/VR enhanced immersive gaming control to provide a positive rehabilitation experience with enhanced engagement and reduced weariness especially for long‐term repeated training, which could largely improve the rehabilitation efficiency by providing timely feedback on the quality of the tasks the users perform. Meanwhile, the smart insole equipped with two TENG sensors is applied to collect diversified gait information for walking status detection. On top of it, a 98.4% identification accuracy for five different users is realized by leveraging ANNs machine learning algorithm to further analyze the signals, aiming at rehabilitation plan selection to ensure the privacy of personal information. Moreover, both the TENG‐based wearable devices are further integrated into the lower‐limb rehabilitation robot (iReGo) and it is demonstrated to achieve multiple functions including user recognition, rehabilitation monitoring, robotic manipulation as well as gaming‐enhanced training. In general, the proposed triboelectric sensory system provides a low‐cost, energy‐saving, and universal solution for the realization of the AIoT‐based smart healthcare system in near future.

## 8 Experimental Section

### Fabrication of the Textile‐Based TENG

The pyramid‐patterned triboelectric layer was made of silicone rubber and it was fabricated through the mold designed by Solidworks 2019 and printed by a 3D printer (M200 Plus, Zortrax) with the filament (Z‐ABS, Zortrax). The required amount of part A and part B of the Ecoflex 00–50 (Smooth‐on) was dispensed into a container (1A:1B by weight) and stirred for 1 min before the material was vacuumed for 3 min to reduce the bubbles. Then, the mixed solution was poured into the 3D‐printed mold followed by a 30 min baking at 70 °C for curing. The PES fabric, a kind of high‐fiber stitched textile, and TPU film were cut into 50 mm × 50 mm, while the conductive textile (i.e., Ni‐fabric) sized 35 mm × 35 mm was prepared to serve as both the electrodes and electrification materials. They were composited together to form 0.4 mm thickness TPU‐coated fabric via electronic iron with about 160 °C, where the hot‐melt TPU film in the middle acted as the adhesive. After gluing the silicone layer onto the Ni‐fabric face of the composite textile with the liquid silicone, a thermal sealer (PFS‐400‐1, Gude) was utilized to bond the TPU film's marginal area to seal the TENG structure.

### Characterization and Electrical Measurement

The open‐circuit voltage, short‐circuit current, and transferred charges were measured by an electrometer (Model 6514, Keithley) and recorded by an oscilloscope (DSOX3034T, Keysight). The measurement for TENG signal optimization was realized by the same oscilloscope with a 100 MΩ impedance probe. The required sensing range for both gait and waist motion detection was estimated by arranging the commercially available sensor (RPS40‐LT, KCUT) onto the safety belt and insole. In order to validate the sensor output for different application scenarios, a multi‐material test system (ZQ‐990, ZHIQU) was accepted to regulate the compressing force and speed. Besides, the TENG‐based sensor was heated by the heating plate (JK‐002, Jiukou) to verify its performance under distinct thermal conditions.

### Sensory System and Applications

Two and four as‐fabricated triboelectric sensors were sewed onto the insole and safety belt, respectively. The real‐time signals for all the applications were collected by the MCU (ATmega328, Arduino UNO) with the signal processing circuit. The commercial Bluetooth module (HC‐05) was utilized to transmit the data in the vehicle control application. The ANN‐based machine learning system was developed in Python with Keras and Tensorflow frameworks. The robot‐aided rehabilitation was conducted by integrating the triboelectric sensory system into the low‐limb rehabilitation robot (iReGo) capable of supporting the user to walk.

### Study Participation

Prior to participation in the experiments, informed consent was obtained from the volunteer in all experiments.

## Conflict of Interest

The authors declare no conflict of interest.

## Supporting information

Supporting Information

Supplemental Video 1

Supplemental Video 2

Supplemental Video 3

Supplemental Video 4

Supplemental Video 5

Supplemental Video 6

Supplemental Video 7

## Data Availability

The data that support the findings of this study are available from the corresponding author upon reasonable request.
