# Supplementary material for: Wearable Triboelectric Sensors Enabled Gait Analysis and Waist Motion Capture for IoT‐Based Smart Healthcare Applications
Source: Adv Sci (Weinh). 2021 Nov 19;9(4):2103694. doi: 10.1002/advs.202103694 (PMC8811828; doi:10.1002/advs.202103694)
Supplement: Supplementary file 1 — Supporting Information [file ADVS-9-2103694-s001.pdf]

© 2021 Wiley-VCH GmbH

ADVANCED SCIENCE
Open Access

Supporting Information

for *Adv. Sci.,* DOI: 10.1002/advs.202103694

Wearable triboelectric sensors enabled gait analysis and waist motion capture for IoT-based smart healthcare applications

Quan Zhang, Tao Jin, Jianguo Cai, Liang Xu, Tianyiyi He, Tianhong Wang, Yingzhong Tian, Long Li, Yan Peng*, Chengkuo Lee**

Supporting Information

Wearable triboelectric sensors enabled gait analysis and waist motion capture for IoT-based smart healthcare applications

Quan Zhang, Tao Jin, Jianguo Cai, Liang Xu, Tianyiyi He, Tianhong Wang, Yingzhong Tian, Long Li, Yan Peng*, Chengkuo Lee**

The file includes:

Figure. S1. Schematics of the fabrication process of the textile-based triboelectric sensor with pyramid structures.

Figure. S2. Testing devices for the optimization of TENG's performance

Figure. S3. Characterization of the TENG sensor with different structures and triboelectric materials.

Figure. S4. SEM images of the aluminum layer and Ni-fabric layer.

Figure. S5. Diagrams of the as-fabricated textile-based TENG devices.

Figure. S6. Characterization of the as-fabricated TENG sensor.

Figure. S7. Output characterizations at different compressing speeds.

Figure. S8. Demonstration of lightening 55 LEDs by the TENG sensor.

Figure. S9. Open-circuit voltages of the TENG at different temperatures.

Figure. S10. Schematic diagram of the circuit to measure the real-time TENG signal.

Figure. S11. Voltages for the vehicle control based on the TENG sensor embedded into the safety belt.

Figure. S12. Characterization of the force on the forefoot during walking.

Figure. S13. Methods and results for measuring real-time walking speed based on TENG-based smart insole, where each step matches the spots on the ground.

Figure. S14. Schematics of the process and parameters for constructing the ANN structure.

Figure. S15. Cross-entropy loss function results for smart insole-based patient recognition with different fully connected layers (FCLs).

Figure. S16. Structure of the rehabilitation robot (iReGo).

Video S1. W-TENG-based vehicle manipulation for waist training

Video S2. Demonstration of walking speed detection based on the smart insole

Video S3. Smart insole-enabled alarm for PD and falling down

Video S4. Demonstration of patient recognition with TENG signals and machine learning.

Video S5. Demonstration of gaming-enhanced waist training

Video S6. Demonstration of iReGo motion control based on TENG sensors

Video S7. Demonstration of robot-aided walking training

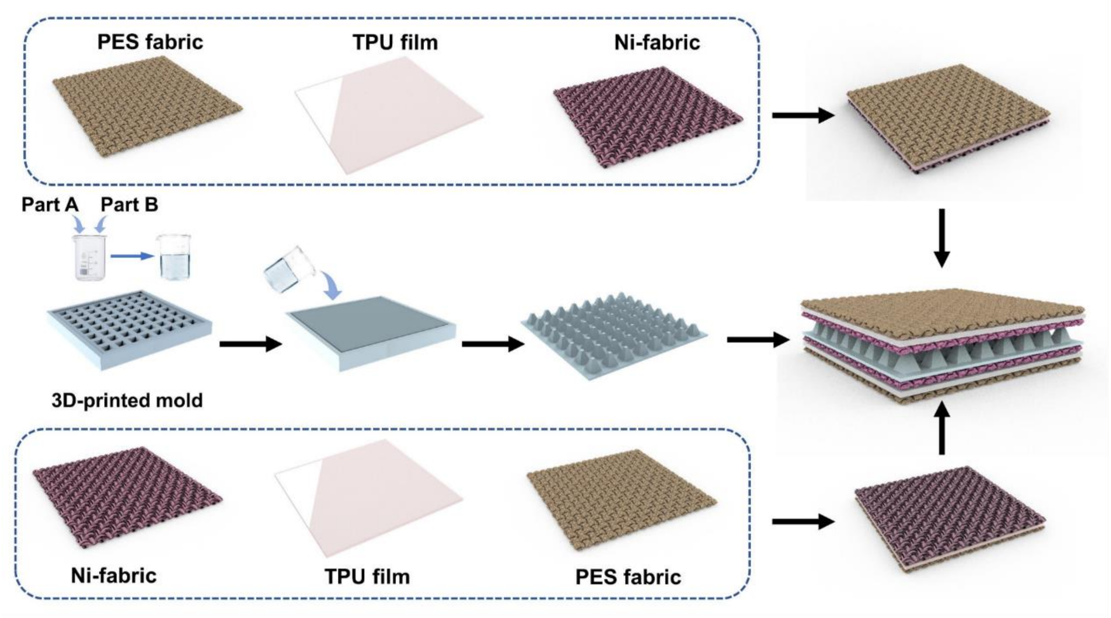

Figure. S1. Schematics of the fabrication process of the textile-based triboelectric sensor with pyramid structures.

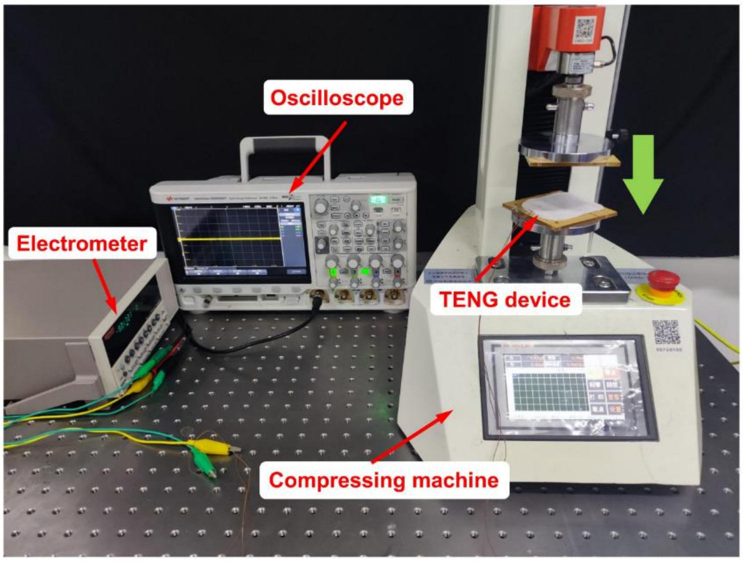

Figure. S2. Testing devices for the optimization of TENG's performance

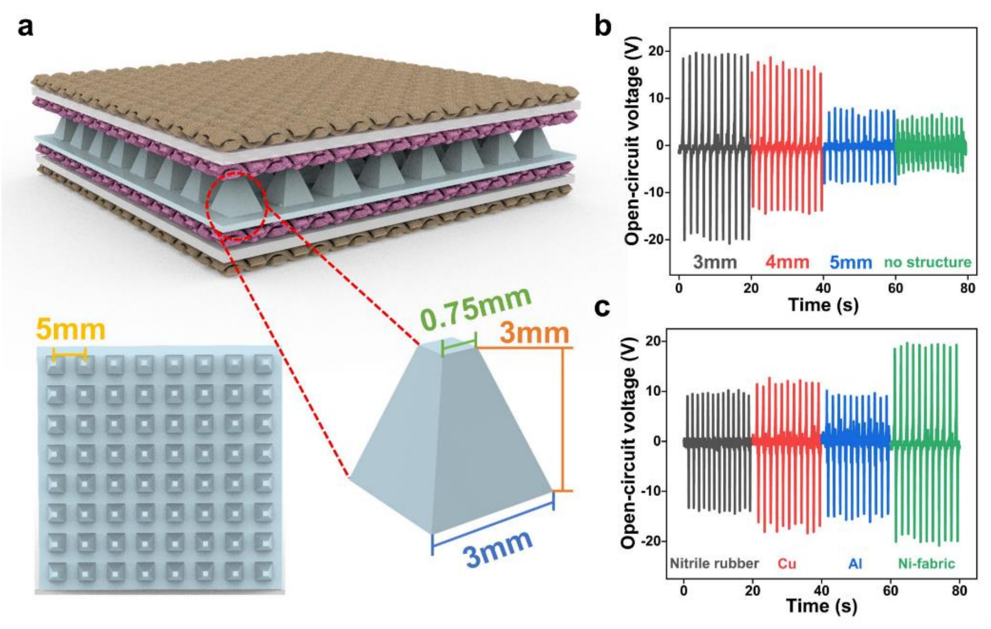

Figure. S3. Characterization of the TENG sensor with different structures and triboelectric materials. **(a)** Dimensions of the pyramid structures. **(b)** Open-circuit voltages of the TENG sensor with different pyramid structures or without pattern under 100 N compressing force and 0.5 Hz frequency. **(c)** Open-circuit voltages of the TENG sensor with different positively triboelectric materials under 100 N compressing force and 0.5 Hz frequency, where pyramid structure is in size of 3 mm.

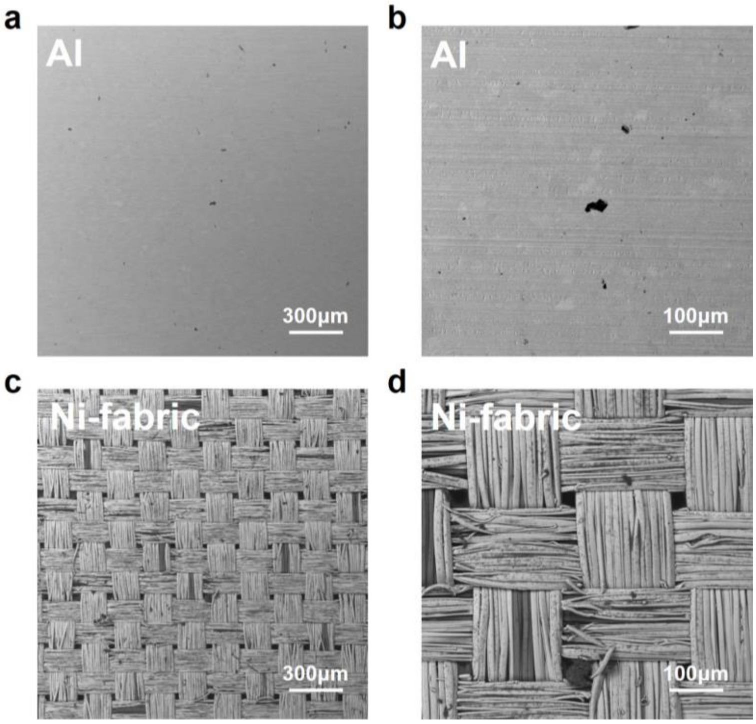

Figure. S4. SEM images of the aluminum layer and Ni-fabric layer. **(a)-(b)** SEM images of aluminum layer with 300 μm and 100 μm. **(c)-(d)** SEM images of Ni-fabric layer with 300 μm and 100 μm.

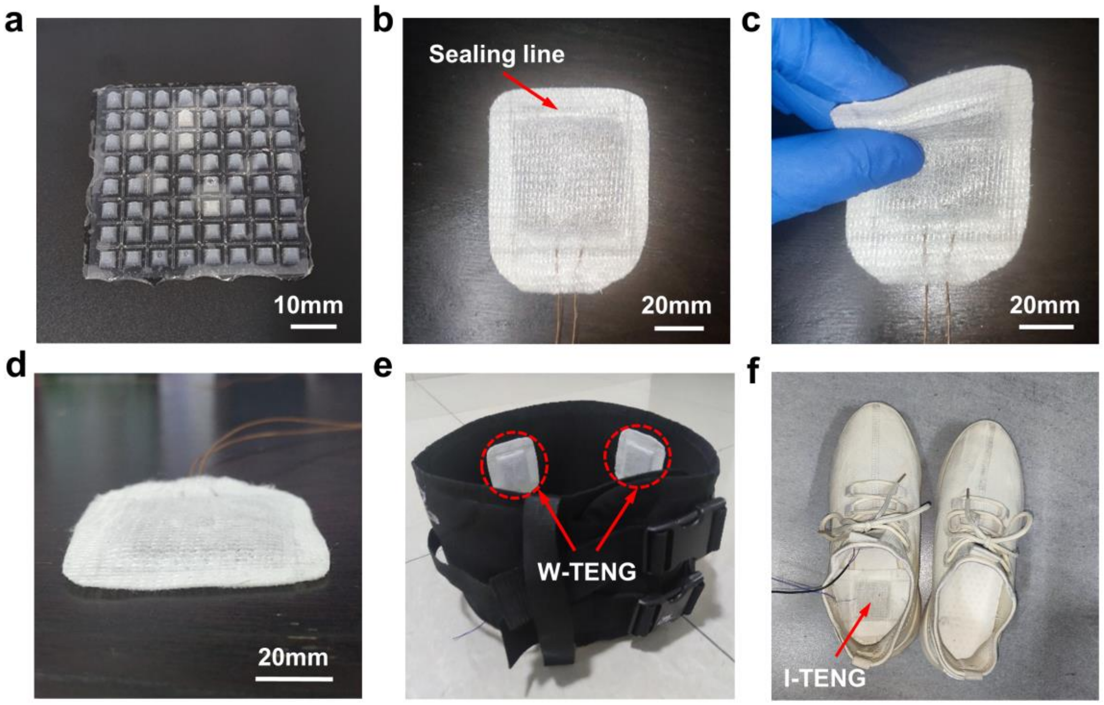

Figure. S5. Diagrams of the as-fabricated textile-based TENG devices. **(a)** Silicone rubber layer with designed pyramid structures. **(b)-(d)** Images of the flexible TENG sensor. **(e)** Image of the as-fabricated safety belt with four TENG sensors. (**f**) Smart insole with TENG sensors for rehabilitation training.

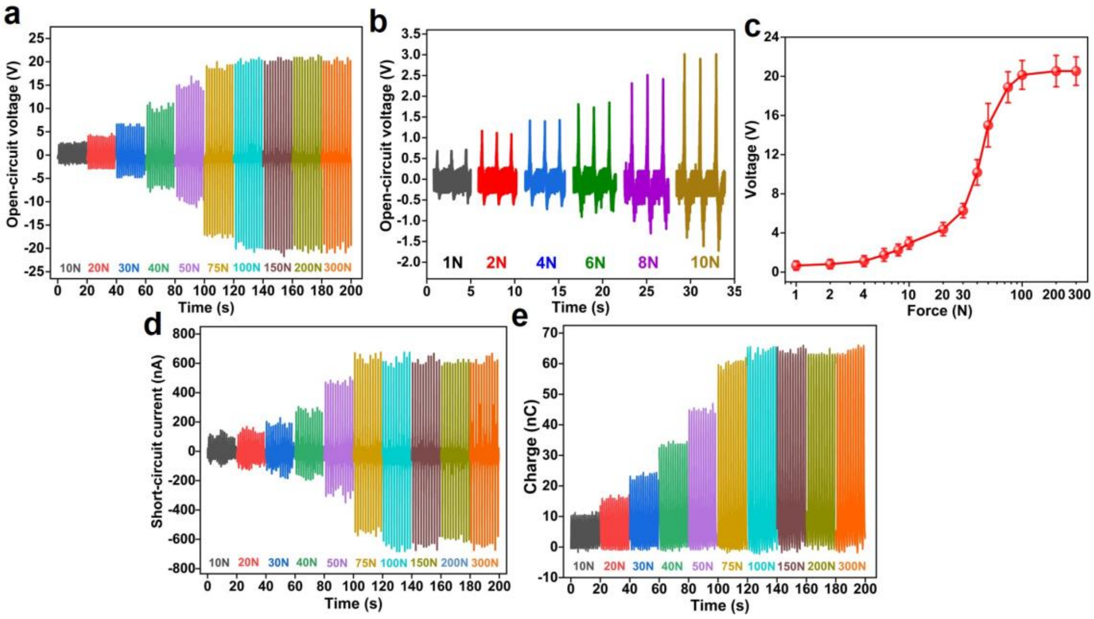

Figure. S6. Characterization of the as-fabricated TENG sensor. **(a)-(b)** Open-circuit voltages of the TENG sensor under different compressing forces. **(c)** Average peak voltage value of five tests. **(d)** Short-circuit currents of the TENG sensor. **(e)** Transferred charges of the TENG sensor.

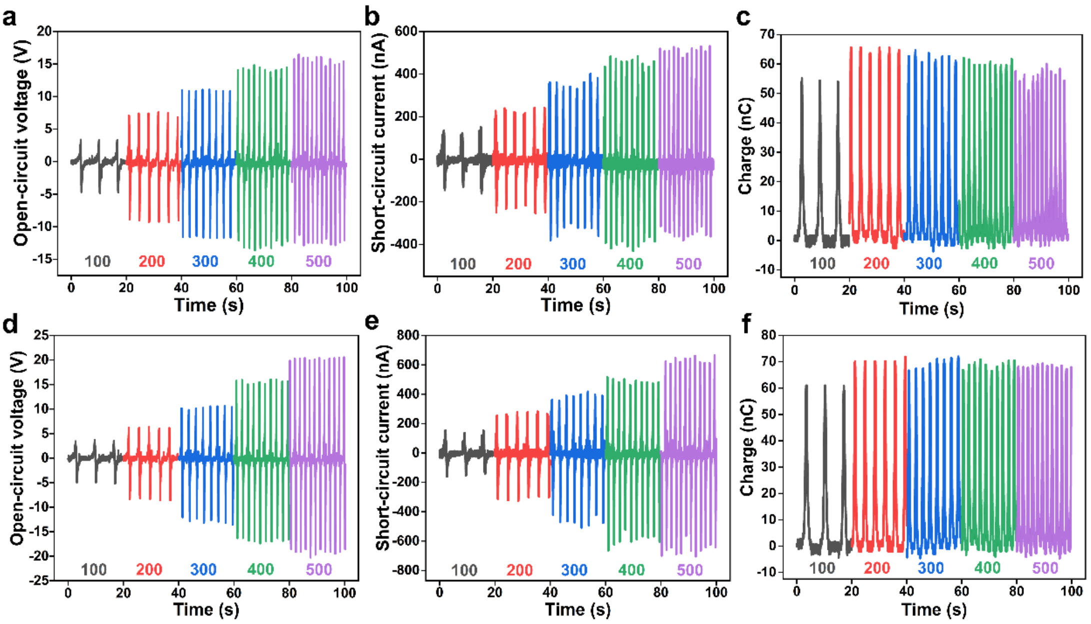

Figure. S7. Output characterizations at different compressing speeds. **(a)-(c)** Open-circuit voltages, short-circuit currents and transferred charges of the TENG sensor with 50 N compressing force at different compressing speeds. **(d)-(f)** Open-circuit voltages, short-circuit currents and transferred charges of the TENG sensor with 100N compressing force at different compressing speeds. The unit of compressing speed in this figure is mm/min.

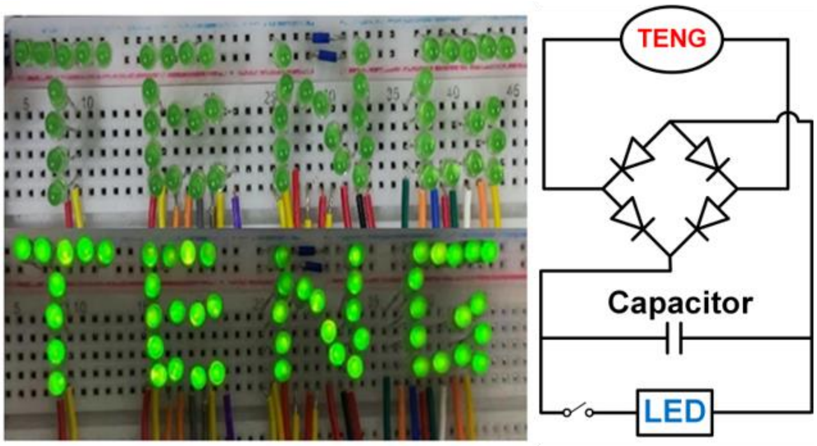

Figure. S8. Demonstration of lightening 55 LEDs by the TENG sensor.

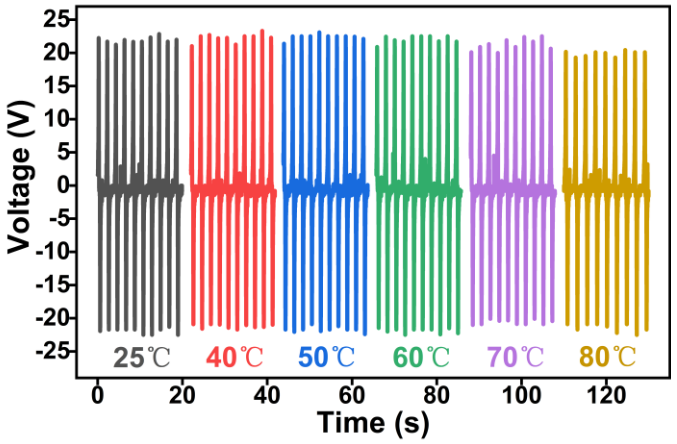

Figure. S9. Open-circuit voltages of the TENG at different temperatures.

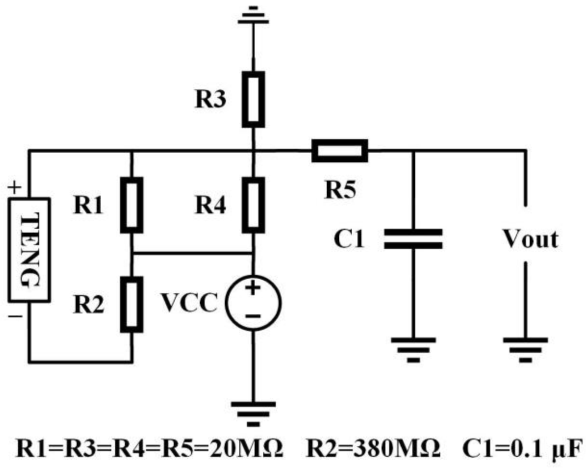

Figure. S10. Schematic diagram of the circuit to measure the real-time TENG signal.

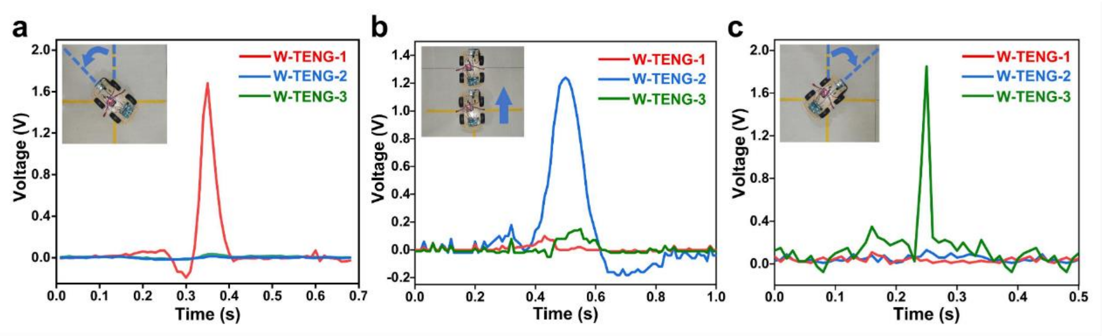

Figure. S11. Voltages for the vehicle control based on the TENG sensor embedded into the safety belt. **(a)-(c)** Turning left, going forward and turning right of the vehicle by compressing W-TENG-1, W-TENG-2, and W-TENG-3 respectively.

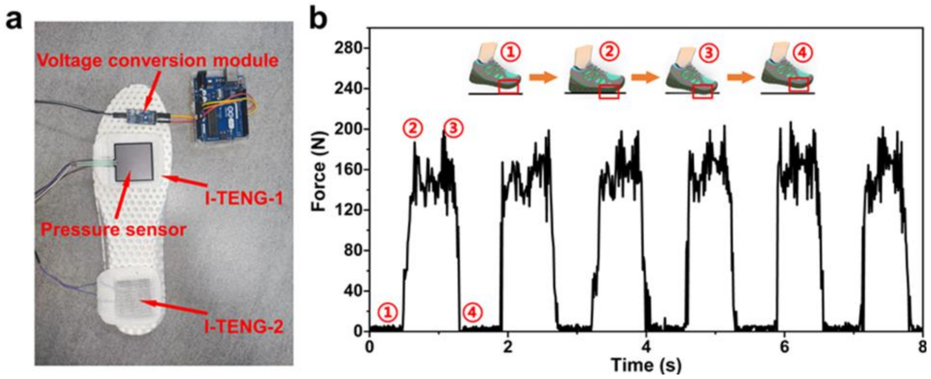

Figure. S12. Characterization of the force on the forefoot during walking. **(a)** Device of the characterization. **(b)** Force values on the forefoot for different periods of walking.

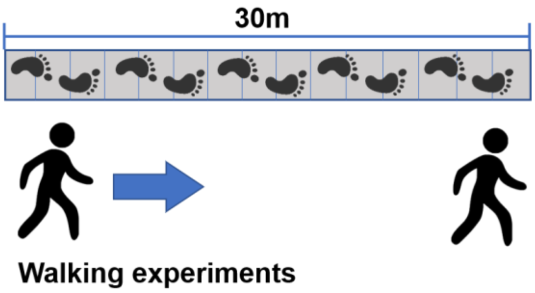

Figure. S13. Methods and results for measuring real-time walking speed based on TENG-based smart insole, where each step matches the spots on the ground.

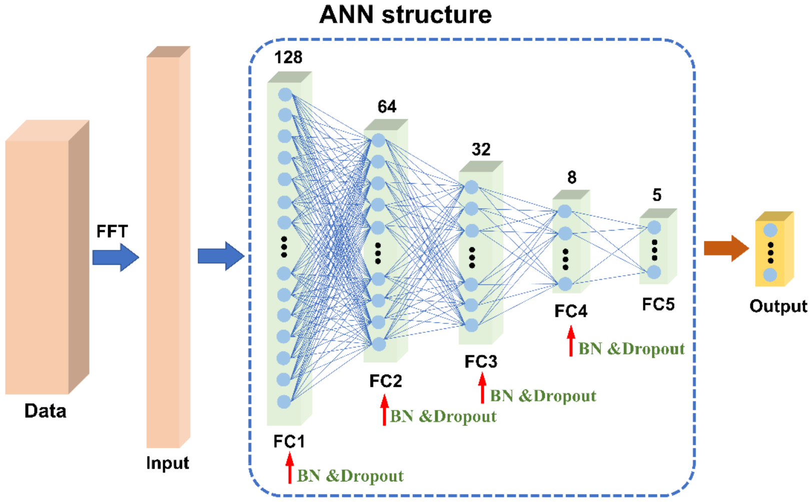

Figure. S14. Schematics of the process and parameters for constructing the ANN structure, where BN represents Batch Normalization.

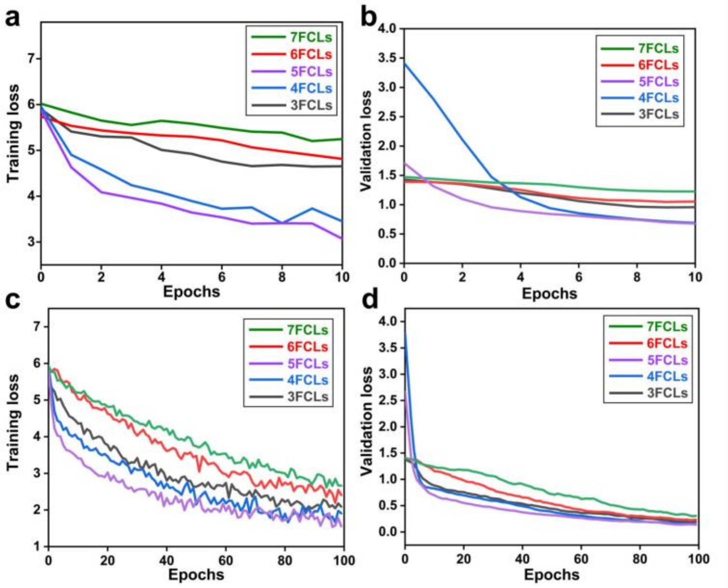

Figure. S15. Cross-entropy loss function results for smart insole-based patient recognition with different fully connected layers (FCLs). (a) The result for training loss and (b) validation loss for 10 epochs of training. (c) The result for training loss and (b) validation loss for 100 epochs of training.

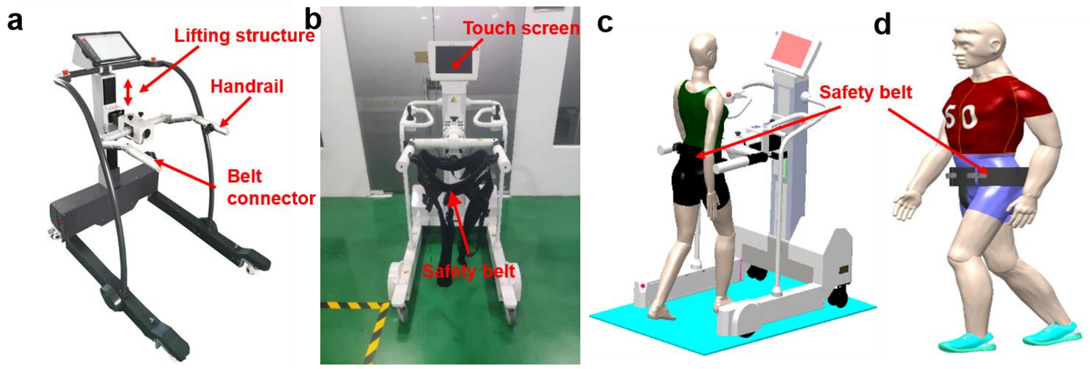

Figure. S16. Structure of the rehabilitation robot (iReGo). **(a)-(b)** Images of iReGo. **(c)** Rehabilitation training with the safety belt to support the patient. **(d)** Schematic of human wearing the TENG-based safety belt and smart insole.
